# Construction Notes

**Published:** 1907-03-02

**Authors:** 


### CONSTRUCTION NOTES.

### ASHTON DISTRICT INFIRMARY.

A large balcony has been added to the upper ward in the Children's Hospital. Another improvement has been the laying out and planting of a piece of waste land adjoining the Kershaw Hospital under the supervision of Mr. Samuel Turner, F.R.H.S. During the year the necessity of an additional room for the treatment of out-patients was felt very strongly by the Medical Board, with the result that the passage adjoining the consulting-room has been widened so as to make a second room, and this has been largely used for eye-testing purposes. The machinery of the laundry has been improved, and is now driven by electricity. The governors have had the drives, court-yard, and approaches to various buildings asphalted, and there is now less noise and less discomfort to patients brought by means of vehicles for treatment at the infirmary.

### SALFORD ROYAL HOSPITAL.

At a largely attended meeting of the Board of Management of the Salford Royal Hospital, the report of the Extension Committee was presented on the recent competition of designs for the enlargement of the hospital. Mr. Keith D. Young, F.R.I.B.A., who was nominated by the London Royal Institute of British Architects to act as assessor, advised that the three premiums of £50, £30, and £20 be awarded to the authors of designs "F," "D," and "E" respectively, and on the recommendation of the Extension Committee this was adopted by the Board. It was announced that the author of design "F" was Mr. John Ely, F.R.I.B.A., of King Street West, Manchester. The plans sent in by the eight competing architects will be publicly exhibited in due course. The Board decided unanimously that the Extension Committee should issue an urgent appeal for £70,000 to enable them to carry out the complete scheme of extension, which includes the purchase of land.

### CHESTERFIELD HOSPITAL.

The Governors of the Chesterfield and North Derbyshire Hospital have approved a scheme for the extension of that hospital, which is to cost £15,000. The main idea of the scheme is to remove the nursing and domestic staffs from the hospital proper and build a nurses' home. The space thus set free will be utilised for the accommodation of patients. The piece of land between the hospital and Infirmary Road, given by Alderman Eastwood, J.P., will be utilised for the staff house. The number on the nursing staff requiring housing will be 47, and accommodation will be provided for 56. Among other features contemplated by the scheme are a mortuary and post-mortem room, electric-light installation, a new surgical ward for 17 men, a new anæsthetic room, the enlargement of the women's ward from 8 to 15 beds, a new ward for 21 children's cots, a new observation ward for three beds, a new ophthalmic ward, and a new $x$-ray room. The changes contemplated will give accommodation for 39 additional in-patients.

### SOUTHEND VICTORIA HOSPITAL.

The water-supply of the hospital has been remodelled and fire hydrants provided. At the last annual meeting it was reported that plans had been prepared and approved for a children's ward to be erected immediately in the rear of the men's ward. Tenders were then got in from local contractors, and that of Messrs. R. Elvy and Son was accepted at £994; to which was added £85 for the removal and rebuilding of the west boundary wall, consequent on the purchase of an additional piece of land 50 feet in width on the west side of the old site. The Committee received an offer from the family of the late Mr. Thomas Dowsett to contribute £1,000, the estimated cost of the children's ward, as a fitting tribute to his memory and in commemoration of his connection with the hospital; in return for which the ward has been named the "Dowsett" ward. For some time past, owing to the increase in the number of patients treated, the medical staff has urged the necessity of enlarging and better equipping the operating theatre and providing an anæsthetic room. A generous offer has been made by Sir Horace Brooks Marshall to equip the theatre with a proper supply of instruments. The Committee accordingly deems it to be an appropriate time to carry out the recommendations of the medical staff, and on plans prepared by Messrs. Greenhalgh and Brockbank, honorary architects, tenders will shortly be invited for the work of enlargement at an estimated cost of £150.

